# Comparison between 1973 and 2004/2016 World Health Organization grading in upper tract urothelial carcinoma treated with radical nephroureterectomy

**DOI:** 10.1007/s10147-021-01941-9

**Published:** 2021-06-06

**Authors:** Claudia Collà Ruvolo, Christoph Würnschimmel, Mike Wenzel, Luigi Nocera, Giuseppe Celentano, Francesco Mangiapia, Zhe Tian, Shahrokh F. Shariat, Fred Saad, Felix H. C. Chun, Alberto Briganti, Nicola Longo, Vincenzo Mirone, Pierre I. Karakiewicz

**Affiliations:** 1 Cancer Prognostics and Health Outcomes Unit, Division of Urology, University of Montréal Health Center, Montreal, QC Canada; 2 Urology Unit, Department of Neurosciences, Reproductive Sciences and Odontostomatology, University of Naples Federico II, Naples, Italy; 3 Department of Urology and Division of Experimental Oncology, URI, Urological Research Institute, IBCAS San Raffaele Scientific Institute, Milan, Italy; 4 Martini-Klinik Prostate Cancer Center, University Hospital Hamburg-Eppendorf, Hamburg, Germany; 5 Department of Urology, University Hospital Frankfurt, Frankfurt am Main, Germany; 6 Department of Urology, Comprehensive Cancer Center, Medical University of Vienna, Vienna, Austria; 7 Departments of Urology, Weill Cornell Medical College, New York, NY USA; 8 Department of Urology, University of Texas Southwestern, Dallas, TX USA; 9 Department of Urology, Second Faculty of Medicine, Charles University, Prag, Czech Republic; 10 Institute for Urology and Reproductive Health, I.M. Sechenov First Moscow State Medical University, Moscow, Russia; 11 Division of Urology, Department of Special Surgery, Jordan University Hospital, The University of Jordan, Amman, Jordan

**Keywords:** WHO, Grade, UTUC, SEER, Pathology

## Abstract

**Aims:**

The European Association of Urology guideline for upper tract urothelial carcinoma (UTUC) relies on two grading system: 1973 World Health Organization (WHO) and 2004/2016 WHO. No consensus has been made which classification should supersede the other and both are recommended in clinical practice. We hypothesized that one may be superior to the other.

**Methods:**

Newly diagnosed non-metastatic UTUC patients treated with radical nephroureterectomy were abstracted from the Surveillance, Epidemiology, and End Results database (2010–2016). Kaplan–Meier plots and multivariable Cox regression models (CRMs) tested cancer-specific mortality (CSM), according to 1973 WHO (G_1_ vs. G_2_ vs. G_3_) or to 2004/2016 WHO (low-grade vs. high-grade) grading systems. Haegerty’s C-index quantified accuracy.

**Results:**

Of 4271 patients, according to 1973 WHO grading system, 134 (3.1%) were G_1_, 436 (10.2%) were G_2_ and 3701 (86.7%) were G_3_; while according to 2004/2016 WHO grading system, 508 (11.9%) were low grade vs 3763 (88.1%) high grade. In multivariable CRMs, high grade predicted higher CSM (Hazard ratio: 1.70, *p* < 0.001). Conversely, neither G_2_ (*p* = 0.8) nor G_3_ (*p* = 0.1) were independent predictors of worse survival. The multivariable models without consideration of either grading system were 74% accurate in predicting 5-year CSM. Accuracy increased to 76% after either addition of the 1973 WHO or 2004/2016 WHO grade.

**Conclusions:**

From a statistical standpoint, either 1973 WHO or 2004/2016 WHO grading system improves the accuracy of CSM prediction to the same extent. In consequence, other considerations such as intra- and interobserver variability may represent additional metrics to consider in deciding which grading system is better.

**Supplementary Information:**

The online version contains supplementary material available at 10.1007/s10147-021-01941-9.

## Introduction

Upper tract urothelial carcinoma (UTUC) is a rare and aggressive malignancy, with an estimated annual incidence in Western Countries of almost two cases per 100,000 inhabitants [1] and with non-organ confined stage in two-third of newly diagnosed patients [2–5]. After stage, tumor grade is the most important predictor of cancer-specific mortality (CSM) in UTUC patients [6–10]. The most recent European Association of Urology (EAU) UTUC guideline relies and recommends the use of two different grading systems. These consist of the 1973 World Health Organization (WHO) and the 2004/2016 WHO classification. Specifically, the 1973 WHO grading system [11] is based on three tiers. Grade 1 applies to tumors with least degree of cellular anaplasia. Grade 3 applies to tumors with most severe degrees of cellular anaplasia. Finally, grade 2 lies in between. Conversely, the 2004/2016 WHO grading system [12, 13] is based on two tiers. It relies on more detailed histological criteria. Low-grade carcinoma applies to tumors with predominantly ordered cell organization with mainly round–oval nuclear shape and mild nuclear chromatin variation. High grade applies to tumors with predominantly disordered cell organization with loss of polarity, moderate to marked nuclear pleomorphism and mainly hyperchromasia [14]. Since there is no consensus on which of the two grading systems should be used in everyday clinical practice [12, 15] and since both are recommended [2], we hypothesized that one may be better. To test this hypothesis, we examined the ability of either the 1973 or the 2004/2016 WHO grading system in predicting CSM, in a contemporary cohort of non-metastatic UTUC patients treated with radical nephroureterectomy (RNU), identified within a large-scale database, namely the Surveillance, Epidemiology and End Results, from 2010 to 2016.

## Materials and methods

### Study population

The 2019-release SEER-18 registry database covers 34.6% of the United States population [16]. Within SEER-18 database (2010–2016), we identified patients aged ≥ 18 year, diagnosed with primary histologically confirmed urothelial carcinoma of renal pelvis or ureter [International Classification of Disease for Oncology (ICD-O-3) site code C65.9 and C.66.9] and treated with RNU. Autopsy and death certificate only cases, with other histology than urothelial (*n* = 142), distant metastases (*n* = 323), unknown T-stage (*n* = 43) and unknown grade (*n* = 813) were excluded. These inclusion criteria yielded 4271 patients.

### Variables definition

Tumor grade was defined according to both the 1973 WHO grading system [grade 1 (G_1_) vs. grade 2 (G_2_) vs. grade 3 (G_3_)] and the 2004/2016 WHO grading system (low grade vs. high grade). Covariables consisted of age, sex, primary site (renal pelvis, ureter), T-stage (T_1_ vs. T_2_ vs. T_3_ vs. T_4_), N-stage (N_0_ vs. N_+_ vs. N_x_) and chemotherapy administration (yes vs. no/unknown). CSM was defined as deaths related to UTUC, according to SEER mortality code [17] and represented the endpoint of interest.

### Statistical analyses

Kaplan–Meier plots and multivariable Cox regression models predicting CSM were fitted. These models relied on T-stage, N-stage, chemotherapy administration and primary site, without including grade. Subsequently, the models were refitted with all previously included variables in addition to the 1973 WHO grading system. Finally, the models were refitted again, this time, with the 2004/2016 WHO grading system. Within Cox models, independent predictor status of WHO grading system was tested. Sensitivity analyses testing the effect of grade (1973 and 2004/2016 WHO grading systems) on CSM were performed in UTUC patients with T_1_ stage and in UTUC patients with T_2_ or lower stage. Finally, the effect of 2004/2016 WHO grading system on CSM was tested in UTUC patients with G_2_ grade, according to the 1973 WHO grading system. Subsequently, accuracy of 5-year CSM predictions was quantified based on multivariable models without consideration of WHO grading system, as well as with consideration of either the 1973 or the 2004/2016 WHO grading system. Haegerty’s C-index quantified accuracy. All statistical tests were two sided, with a level of significance set at *p* < 0.05. Statistical analyses were performed using the R software environment for statistical computing and graphics, version 4.0.0 (available at: http://www.rproject).

## Results

### Descriptive characteristics

**Fig. 1:**
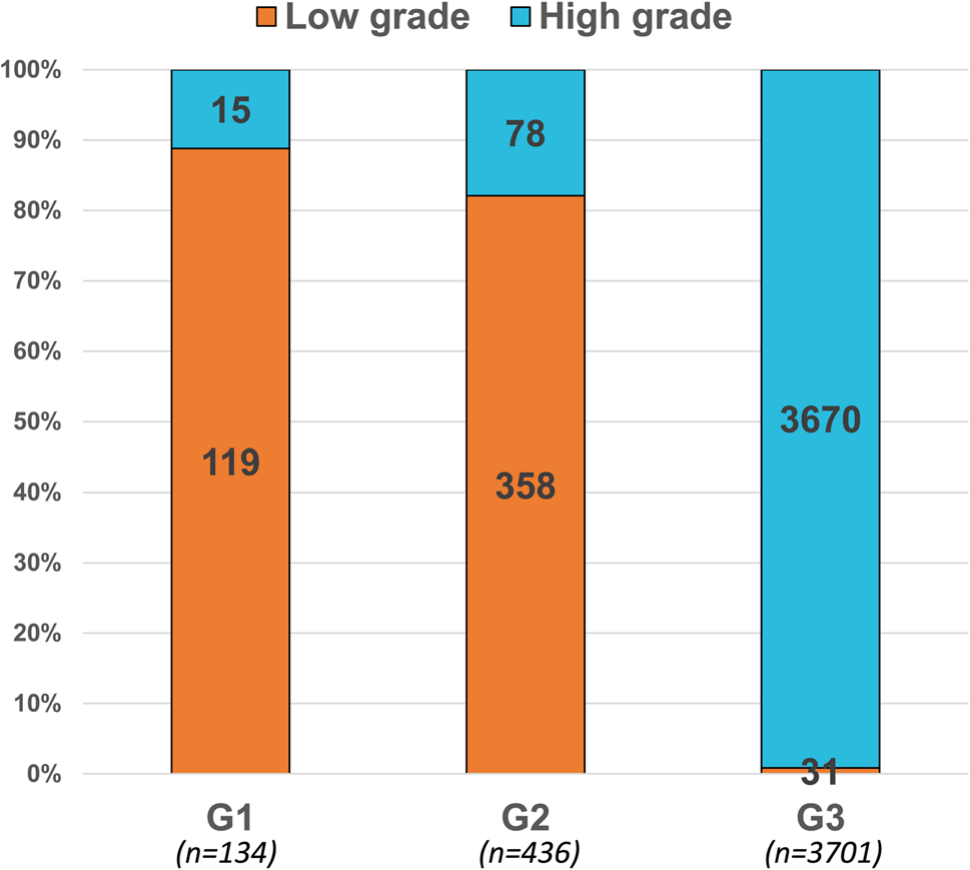
Stacked barplot depicting the rates of tumor grade according to the 2004/2016 WHO grading system (low grade vs high grade) in 134, 436 and 3701 G1, G2 and G3 non-metastatic upper tract urothelial carcinoma patients treated with radical nephroureterectomy, according to the 1973 WHO grading system, respectively

From 2010 to 2016, 4271 cases of UTUC treated with RNU were identified (Table 1). Of those, according to 1973 WHO grading system, 134 (3.1%) were G_1_, 436 (10.2%) were G_2_ and 3701 (86.7%) were G_3_; while according to 2004/2016 WHO grading system, 508 (11.9%) were low grade vs 3763 (88.1%) high grade. The median age was 73 years (Interquartile range: 65–80). Most patients were male (*n* = 2575, 60.3%), with renal pelvis urothelial carcinoma (*n* = 2906, 68.0%) and harbored T_3_ stage at RNU (*n* = 1867, 43.7%). Finally, 897 (21.0%) patients received chemotherapy. Of all G_1_ patients (*n* = 134), 119 (88.8%) and 15 (11.2%) were low grade and high grade, respectively. Of all G_2_ patients (*n* = 436), 358 (82.1%) and 78 (17.9) were low grade and high grade, respectively. Of all G_3_ patients (*n* = 3701), 31 (0.8%) and 3670 (99.2%) were low grade and high grade, respectively (Fig. 1).

**Table 1:** Baseline characteristics of 4271 upper tract urothelial carcinoma patients treated with radical nephroureterectomy, identified within Surveillance, Epidemiology and End Results database, between 2010 and 2016

Overall, *n* (%)	4271 (100)
1973 WHO grading system, *n* (%)	
G_1_	134 (3.1)
G_2_	436 (10.2)
G_3_	3701 (86.7)
2004/2016 WHO grading system, *n* (%)	
Low grade	508 (11.9)
High grade	3763 (88.1)
Age	
Median	73
IQR	65–80
Sex, *n* (%)	
Female	1696 (39.7)
Male	2575 (60.3)
Follow-up	
Median	22
IQR	10–43
Primary site, *n* (%)	
Renal pelvis	2906 (68.0)
Ureter	1365 (32.0)
T-stage, *n* (%)	
T_1_	1306 (30.6)
T_2_	747 (17.5)
T_3_	1867 (43.7)
T_4_	351 (8.2)
N-stage, *n* (%)	
N_0_	3711 (86.9)
N_+_	476 (11.1)
N_x_	84 (2.0)
Chemotherapy, *n* (%)	
No/unknown	3374 (79.0)
Yes	897 (21.0)

*IQR* interquartile range, *WHO* World Health Organization

### Survival analyses and accuracy in predicting CSM

**Fig. 2:**
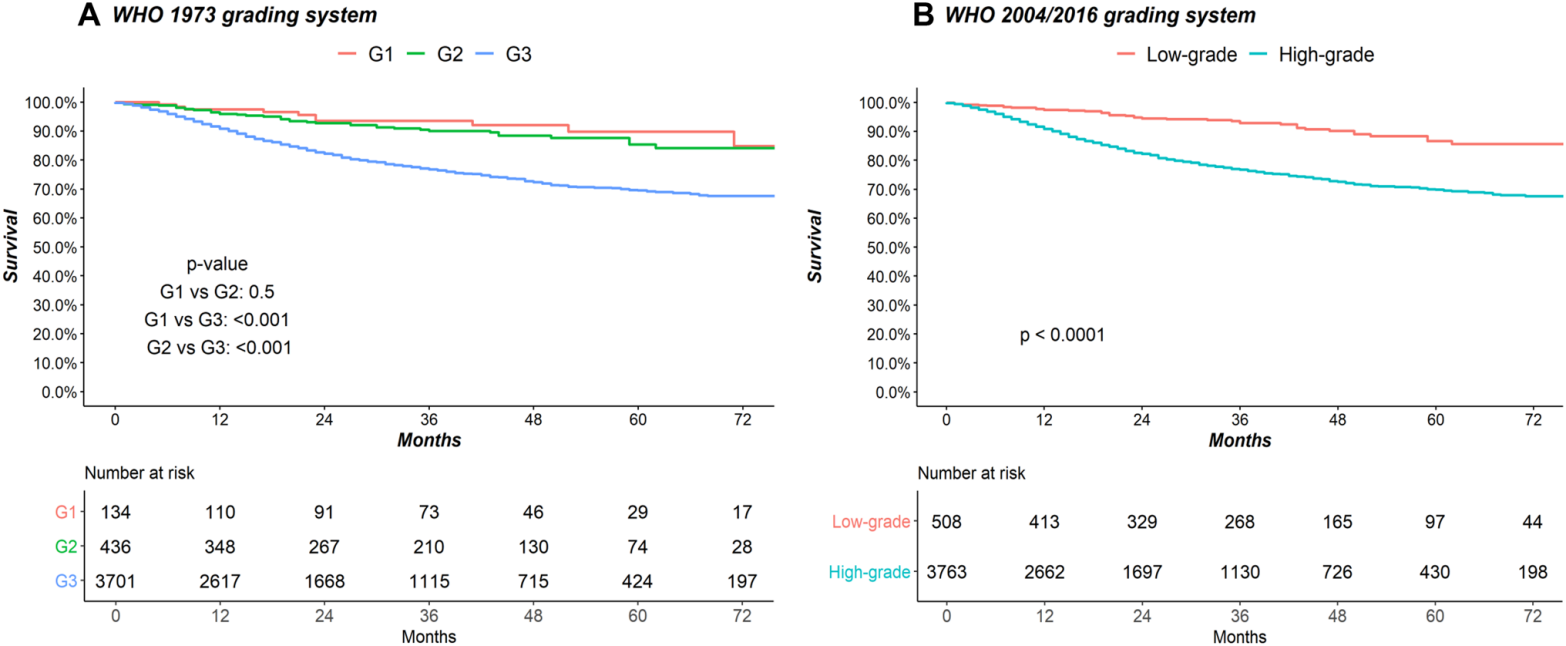
Kaplan–Meier plots depicting cancer-specific mortality (CSM) in 4271 non-metastatic upper tract urothelial carcinoma patients treated with radical nephroureterectomy, identified within Surveillance, Epidemiology and End Results (2010–2016), according to the **A** 1973 World Health Organization (WHO) grading system and to the **B** 2004/2016 WHO grading system

In overall population, according to 1973 WHO grading system (Fig. 2A), 5-year CSM rates were 10.2%, 14.6% and 30.5% for G_1_, G_2_ and G3 UTUC grade, respectively. In multivariable Cox regression models focusing on CSM (Table 2), relative to G_1_, neither G_2_ [Hazard ratio (HR) 1.07, *p* = 0.8] or G_3_ (HR 1.65, *p* = 0.1) represented independent predictors. When sensitivity analyses were performed (Supplementary Table 1), the results were confirmed in the multivariable Cox regression models focusing on CSM in patients with T_1_ stage (relative to G_1_, G_2_: HR 1.00, *p* = 1.0 and G_3_: HR 1.82, *p* = 0.2) and T_2_ or lower stage (G_2_ HR: 0.99 *p* = 0.9, G_3_ HR 1.38, *p* = 0.4, relative to G_1_). The accuracy of the multivariable model (Table 4) that included 1973 WHO grading system was 76%. Conversely, the accuracy of the multivariable model without consideration of 1973 WHO grading system was 74%.

**Table 2:** Multivariable Cox regression models predicting cancer-specific mortality (CSM) in 4271 upper tract urothelial carcinoma patients identified within Surveillance, Epidemiology and End Results database (2010–2016), where pathological grade was defined according to the three-tier 1973 World Health Organization (WHO) grading system

	CSM	
	HR (95% CI)	*p*-value
WHO 1973 grading system, relative to G1		
G_2_	1.07 (0.55–2.08)	0.8
G_3_	1.65 (0.90–3.01)	0.1
T-stage, relative to T1		
T_2_	1.85 (1.39–2.48)	< 0.001
T_3_	3.64 (2.88–4.60)	< 0.001
T_4_	10.69 (8.06–14.17)	< 0.001
N-stage, relative to N_0_		
N_+_	1.82 (1.49–2.22)	< 0.001
N_X_	0.79 (0.39–1.59)	0.5
Chemotherapy administration, relative to no/unknown		
Yes	0.80 (0.67–0.96)	0.01
Primary site, relative to renal pelvis		
Ureter	1.19 (1.01–1.40)	0.03

In overall population, according to 2004/2016 WHO grading system (Fig. 2B), 5-year CSM rates were 13.4% and 30.2% for low grade and high grade, respectively. In multivariable Cox regression models focusing on CSM (Table 3), relative to low grade, high grade (HR 1.70, *p* < 0.001) achieved independent predictor status. When sensitivity analyses were performed (Supplementary Table 1), the results were confirmed in the multivariable Cox regression models focusing on CSM in patients with T_1_ stage (relative to low grade, high grade: HR 1.76, *p* = 0.04), T_2_ or lower stage (relative to low grade, high grade: HR 1.65, *p* = 0.02) and G_2_ grade (relative to low grade, high grade: HR 2.19, *p* = 0.02). The accuracy of the multivariable model (Table 4) that included 2004/2016 WHO grading system was 76%. Conversely, the accuracy of the multivariable model without consideration of 2004/2016 WHO grading system was 74%.

**Table 3:** Multivariable Cox regression models predicting cancer-specific mortality (CSM) in 4271 upper tract urothelial carcinoma patients identified within Surveillance, Epidemiology and End Results database (2010–2016), where pathological grade was defined according to the two-tier 2004/2016 World Health Organization (WHO) grading system

	CSM	
	HR (95% CI)	*p*-value
WHO 2004/2006 grading system, relative to low grade		
High grade	1.70 (1.23–2.35)	0.001
T-stage, relative to T_1_		
T_2_	1.84 (1.38–2.46)	< 0.001
T_3_	3.61 (2.86–4.56)	< 0.001
T_4_	10.51 (7.92–13.94)	< 0.001
N-stage, relative to N_0_		
N_+_	1.82 (1.49–2.23)	< 0.001
N_X_	0.79 (0.39–1.60)	0.5
Chemotherapy administration, relative to no/unknown		
Yes	0.80 (0.67–0.96)	0.01
Primary site, relative to renal pelvis		
Ureter	1.19 (1.01–1.40)	0.04

**Table 4:** Accuracy in cancer-specific mortality prediction at 5 years after treatment, in 4217 upper tract urothelial carcinoma patients treated with radical nephroureterectomy, identified within Surveillance Epidemiology and End Results database (2010–2016), based on multivariable Cox models: (1) without grade consideration, (2) considering the three-tier 1973 WHO grading system and (3) considering the two-tier 2004/2016 WHO grading system

	Heagerty’s C-index
(1) Model based on primary site, T-stage, N-stage and chemotherapy administration	0.74
(2) Model based on primary site, T-stage, N-stage, chemotherapy administration with the three-tier WHO 1973 grading classification system	0.76
(3) Model based on primary site, T-stage, N-stage, chemotherapy administration with the two-tier WHO 2004/2016 grading classification system	0.76

*WHO* World Health Organization, *C-index* concordance index

## Discussion

To date, the EAU UTUC guideline relies and recommends the use of two different grade classification system: 1973 WHO and 2004/2016 WHO grading system. Which system should be used in everyday clinical practice is still under debate. We hypothesized that one may be better. To test this hypothesis, we examined the ability of either the 1973 or the 2004/2016 WHO grading system in predicting CSM, in a cohort of non-metastatic UTUC patients treated with RNU. Our analyses showed several noteworthy observations.

First, of all RNU patients examined in the current study (*n* = 4271), approximately 90% harbored the highest grade level, regardless of which grading system was used. Specifically, 86.7% harbored G_3_ according to 1973 WHO grading system and 88.1% harbored high grade according to 2004/2016 WHO grading system. These elevated rates of high-grade UTUC may be explained by the nature of the study population. Specifically, all patients harbored stage T_1_ or higher [18]. Moreover, all patients were treated with RNU. In consequence, a selection bias towards higher grade was operational, relative to studies that also included non-invasive (stages T_a_ and T_is_) UTUC patients treated with less definitive modalities than RNU [19–22]. However, even in those studies, the rate of non-invasive UTUC represented a marginal fraction of the overall population and the vast majority also harbored high-grade disease. For example, Singla et al. [21] examined 753 UTUC patients treated with RNU or distal ureterectomy, between 1998 and 2015. Of those, 78.8% harbored T_1_ or higher stages and 89.2% harbored high-grade UTUC. Moreover, Roupret et al. [22] recorded T_1_ or higher stages in 66 (68.0%) patients and high grade in 50 (51.5%) patients, within 97 UTUC patients, despite ureteroscopy or percutaneous endoscopy treatment.

Second, the current analyses demonstrated marginal discrimination between G_1_ and G_2_, with respect to CSM. Within the three-tier grading system, independent predictor status of G_2_ and G_3_, relative to G_1_, could not be established. These results were confirmed in RNU patients with T_1_ or T_2_ or lower stages. The combination of these observations suggested limited discrimination ability of the three-tier grading system. Nonetheless, the addition of the 1973 WHO grading system resulted in a 2% accuracy gain, relative to multivariable models without consideration of the three-tier grading system. However, a 2% gain may be considered marginal. Specifically, this figure implies that within a cohort of 1000 individuals, the use of the three-tier grading system would improve CSM prediction in 20 patients. This gain is important in large-scale prospective trials or in large-scale epidemiological analyses. However, a 2% gain in predictive accuracy may not be clinically meaningful in everyday clinical practice.

In the second part of the analyses, we focused on the two-tier WHO grading system. Here, we validated the independent predictor status of high grade relative to low grade. Specifically, high-grade UTUC had 1.70-fold, 1.76-fold, 1.65-fold, and 2.19-fold higher risk of CSM, relative to low-grade UTUC in overall population, in T_1_, T_2_ or lower and G_2_ patients, respectively. Finally, we also recorded a 2% accuracy gain, when the 2004/2016 WHO grading system was added to multivariable model, where grade was previously not considered. In consequence, based on accuracy, the added benefit of the 2004/2016 WHO grading system was exactly the same as for the 1973 WHO grading system. However, the discrimination of CSM rates appeared more practical with the two-tier grading system, where high-grade patients exhibited a nearly twofold higher CSM rate and reached independent predictor status. In consequence, it appears that based on statistical criteria used in the current analyses, the two-tier grading system benefits of a slight advantage over its three-tier counterpart.

Additional consideration may be required to decide which grading system should be included in everyday clinical practice and which may be abandoned. Several investigators compared intra- and interobserver variability of the two- vs three-tier grading system in bladder cancer [12, 23–29]. Unfortunately, such analyses did not focus on UTUC. However, based on methodological considerations, a system that relies on two tiers is invariably more likely to result in a lower intra- and interobserver variability than a system with more than two levels. This notion rests on the effect of chance. In consequence, based on similar predictive accuracy, superiority of discrimination in univariable and multivariable models, and on methodological consideration of intra- and interobserver variability, it appears that the two-tier grading system might represent a better alternative. However, specific expert intra- and interobserver variability testing in UTUC patients should ideally complement the findings of our study.

To the best of our knowledge, we are the first to examine the ability of either 1973 or 2004/2016 WHO grading classification in predicting CSM, in UTUC patients identified within a large-scale population-based database. Only one group of investigators [30] examined grade assignment differences according to 1973 vs. 2004/2016 grading system in a smaller cohort (*n* = 458) of UTUC patients treated with RNU, at a single Chinese institution, between 2008 and 2013. Unfortunately, the complexity of the methodology used by Guan et al. renders comparisons with our methodology practically impossible.

Our work is not devoid of limitations and should be interpreted in the context of its retrospective and population-based design. First, the SEER database focuses on invasive UTUC, since T_is_ and T_a_ patients are not included. In consequence, our observations are based on more advanced stage and grade distribution and are not directly comparable with studies that used the entire UTUC population as reference. However, T_is_ and T_a_ patients should ideally not be treated with RNU. In consequence, their exclusion from SEER database does not represent an important limitation for studies that focus on RNU. Second, disease progression or disease recurrence data are not available in the SEER database. In consequence, they cannot be examined as endpoints. Third, the SEER database does not allow to ascertain either type or duration of chemotherapy. Fourth, due to the short median follow-up, future studies with longer follow-up should be done to confirm or refuse our results. Fifth, our study did not benefit of central pathology review. Sixth, our analyses could not assess intra- and interobserver variability, which are essential in clinical practice. Finally, the SEER database represents a proportion of the United States populations. In consequence, our findings are only applicable to patients from the United States and are not be generalizable to patients from other parts of the world. However, these limitations apply to this and to all other studies based on the SEER database.

## Conclusion

From a statistical standpoint, either 1973 WHO or 2004/2016 WHO grading system improves the accuracy of CSM prediction to the same extent. In consequence, other considerations such as intra- and interobserver variability may represent additional metrics to consider in deciding which grading system is better.

## Supplementary Information

Below is the link to the electronic supplementary material.

Supplementary file 1 (DOCX 18 KB)
