# Supplementary material for: Comparison between 1973 and 2004/2016 World Health Organization grading in upper tract urothelial carcinoma treated with radical nephroureterectomy
Source: Int J Clin Oncol. 2021 Jun 6;26(9):1707–13. doi: 10.1007/s10147-021-01941-9 (PMC8364897; doi:10.1007/s10147-021-01941-9)
Supplement: Supplementary file 1 — (DOCX 18 KB) [file 10147_2021_1941_MOESM1_ESM.docx]

|  | **A) T_1_ stage** | | **B) T_2_ or lower stage** | | **C) G_2_ grade** | |
| --- | --- | --- | --- | --- | --- | --- |
|  | ***HR (95% CI)*** | ***p-value*** | ***HR (95% CI)*** | ***p-value*** | ***HR (95% CI)*** | ***p-value*** |
| ***WHO 1973 grading system,***  ***relative to G_1_*** | | | | | | |
| **G_2_** | 1.00 (0.35-2.84) | 1.0 | 0.99 (0.44-2.22) | 0.9 | - | - |
| **G_3_** | 1.82 (0.72-4.53) | 0.2 | 1.38 (0.67-2.83) | 0.4 | - | - |
| ***WHO 2004/2016 grading system,***  ***relative to low-grade*** | | | | | | |
| **High-grade** | 1.76 (1.02-3.04) | 0.04 | 1.65 (1.07-2.55) | 0.02 | 2.19 (1.10-4.36) | 0.02 |
